# Relationship between Early Inflammatory Response and Clinical Evolution of the Severe Multiorgan Failure in Mechanical Circulatory Support-Treated Patients

**DOI:** 10.1155/2014/281790

**Published:** 2014-07-14

**Authors:** Raffaele Caruso, Jonica Campolo, Alessandro Verde, Luca Botta, Lorena Cozzi, Marina Parolini, Filippo Milazzo, Sandra Nonini, Luigi Martinelli, Roberto Paino, Paolo Marraccini, Maria Frigerio

**Affiliations:** ^1^CNR Institute of Clinical Physiology, CardioThoracic and Vascular Department, Niguarda Cà Granda Hospital, Piazza Ospedale Maggiore 3, 20162 Milan, Italy; ^2^CardioThoracic and Vascular Department, Niguarda Cà Granda Hospital, Piazza Ospedale Maggiore 3, 20162 Milan, Italy; ^3^CNR Institute of Clinical Physiology, Via Moruzzi 1, 56124 Pisa, Italy

## Abstract

*Background*. The mechanical circulatory support (MCS) is an effective treatment in critically ill patients with end-stage heart failure (ESHF) that, however, may cause a severe multiorgan failure syndrome (MOFS) in these subjects. The impact of altered inflammatory response, associated to MOFS, on clinical evolution of MCS postimplantation patients has not been yet clarified. *Methods*. Circulating cytokines, adhesion molecules, and a marker of monocyte activation (neopterin) were determined in 53 MCS-treated patients, at preimplant and until 2 weeks. MOFS was evaluated by total sequential organ failure assessment score (tSOFA). *Results*. During MCS treatment, 32 patients experienced moderate MOFS (tSOFA < 11; A group), while 21 patients experienced severe MOFS (tSOFA ≥ 11) with favorable (B group) or adverse (*n* = 13, C group) outcomes. At preimplant, higher values of left ventricular ejection fraction (LVEF) and estimated glomerular filtration rate (eGFR) were the only parameter independently associated with A group. In C group, during the first postoperative week, high levels of interleukin-8 (IL-8) and tumor necrosis factor (TNF)-*α*, and an increase of neopterin and adhesion molecules, precede tSOFA worsening and exitus. *Conclusions*. The MCS patients of C group show an excessive release to IL-8 and TNF-*α*, and monocyte-endothelial activation after surgery, that might contribute to the unfavourable evolution of severe MOFS.

## 1. Introduction

The mechanical circulatory support (MCS) device implantation has emerged as an alternative treatment strategy in critical ill patients with advanced heart failure (HF) [1]. The MCS devices are mechanical pumps that supplement or replace the function of a damaged left ventricle in order to maintain appropriate blood flow among patients with end-stage HF (ESHF). In addition to supporting circulation, MCS, in particular left ventricular assist devices, can lead to other cardiac benefits, such as improved contractility, reduction of hypertrophy, and reversal of chamber enlargement [2]. Left ventricular assist devices are mainly used as a bridge to transplant, with the aim of increasing patient survival until an appropriate organ becomes available [3].

Overall, in critically ill patients, the negative impact of chronic comorbidity on survival is primarily influenced by the degree of multiple organ failure syndrome (MOFS) or the cumulative severity of multiple comorbidities [4]. Indeed, despite advances in technology and subsequent improvements in morbidity and mortality of patients undergoing MCS placement, postimplantation MOFS, and infections remain the major causes of death in these patients [5, 6]. The MOFS seems to be influenced by the degree of the immune-inflammatory response independently of the presence of infection. In MCS-treated patients, liver dysfunction was shown to be associated to the progressive release of inflammatory mediators, such as interleukin-6 (IL-6), IL-8, and C-reactive protein (CRP) [7]. Moreover, platelets and monocytes activation is associated with different MCS devices suggesting a role of their interaction in the development of haematic complications [8]. However, the impact of inflammatory response on MOFS development after MCS implant still needs to be elucidated.

The aim of this study was to investigate, in MCS-treated patients, the early inflammatory signals associated to different MOFS degrees and to their clinical evolution.

## 2. Materials and Methods

### 2.1. Population and Study Design

We included in the study 53 patients with ESHF, not amenable to recovery by pharmacological or conventional surgical therapy, who underwent MCS, according to guideline indications for mechanical support [9].

The hemodynamic parameters, cardiac index (CI), pulmonary capillary wedge pressure (PCWP), and right atrial pressure were measured by pulmonary artery Swan-Ganz catheter. Left ventricular ejection fraction (LVEF) was quantified by transesophageal echocardiography.

We calculated the total sequential organ failure assessment score (tSOFA) according to Pätilä et al. [10] as the highest SOFA value from preimplant to two weeks after surgery. This score is a measure of MOFS development and risk of mortality in the intensive care unit after cardiac surgery. The SOFA is a six-organ (respiration, coagulation, liver, neurological, cardiovascular, and renal) dysfunction/failure score measuring multiple organ failure daily. Each organ is graded from 0 (normal) to 4 (the most abnormal), providing a daily score of 0 to 24 points.

In sedated patients, the neurological score was computed retrospectively, when sedatives were stopped, alternatively, after their temporary discontinuation. Renal function was assessed by estimated glomerular filtration rate (eGFR) using the abbreviated formula [11].

This study complied with the principles of the Declaration of Helsinki. The study protocol has been approved by the Ethics Committee of Niguarda Cà Granda Hospital (Milan, Italy) and a signed informed consent has been obtained by all participating patients.

Peripheral blood samples for biochemical assessment were collected in fasting condition preimplant and after 4 hours 1 day, 3 days, 7 days, and 14 days from MCS.

### 2.2. Biochemical Assessment

Plasma and serum samples were obtained after blood centrifugation at 4000 rpm for 10 minutes.

Plasma interleukin-6 (IL-6), IL-8, IL-10, tumor necrosis factor (TNF)-*α*, IL-1 receptor antagonist (IL-1ra), soluble platelet selectin (sP-selectin), and soluble intercellular adhesion molecule type 1 (sICAM-1) were measured using specific enzyme-linked immunoassays kits (R&D Systems, Minneapolis, MN-USA for IL-6, IL-8, IL-10, IL-1ra, sP-selectin, and sICAM-1; Cayman, Ann Arbor, MI-USA for TNF-*α*) that allow an easy and accurate quantification based on antibody reaction.

Serum CRP levels were evaluated by high-sensitive immune-turbinometric assay (Roche Diagnostic GmbH) which use an anti-CRP monoclonal antibody immobilized on latex particles.

Urinary neopterin levels were measured by an isocratic HPLC method, previously reported [12], and normalized by urine creatinine concentrations. Briefly, urine samples were adequately diluted with chromatographic mobile phase (15 mM of K_2_HPO_4_, pH 3.0). Neopterin and creatinine levels were assessed using a Kontron instrument (pump 422-S, autosampler 465) coupled to a fluorometric detector (JASCO FP-1520, *λ*_ex_ = 355 nm and at *λ*_em_ = 450 nm) for neopterin detection and to a UV-VIS detector (BIO-RAD 1706, *λ* = 240 nm) for creatinine determination. Neopterin and creatinine separations were performed at 50°C on a 5 *μ*m Discovery C18 analytical column (250 × 4.6 mm I.D., Supelco, Sigma-Aldrich) at flow rate of 0.9 mL/min.

### 2.3. Statistical Analysis

Data are presented as median and interquartile range (25th–75th) or frequency. Retrospectively, patients were categorized into three groups: A, B, and C, according to severity grade of MOFS and 3-month outcome. Comparison among groups was conducted by a one-way analysis of variance, or Kruskall-Wallis in case of skewed variables, with Bonferroni post hoc test for continuous variables and by a chi-square test for categorical variables. Multivariable ordinal logistic regression models were constructed to examine the effect of biochemical and clinical parameters with group of MOFS severity as the dependent variable. Results are presented as odds ratio (OR) and their 95% confidence interval (CI). Differences across time within patient groups were assessed by nonparametric Friedman test.

A two-tailed *P* value <0.05 was considered statistically significant. All statistical tests were done with SPSS version 17.0 software (SPSS, Inc., Chicago, IL, USA).

## 3. Results

Clinical data of candidates to MCS implantation and operative characteristics are described in Table 1. Patient age ranged from 22 to 72 years (median 54 (48–61)). Thirty patients had idiopathic dilated cardiomyopathy, twenty-two ischemic cardiomyopathies, and one acute myocarditis. At preimplant, 37 patients were receiving inotropes. Median tSOFA score was 5 (3–7).

In 49 patients intrathoracic left ventricular assist devices were implanted (48 continuous flow pumps (8 DeBakey, 6 Incor, 32 HeartMateII, 2 HeartWare), and 1 pulsatile-flow pump (Novacor)) while in 4 patients an extracorporeal continuous-flow, centrifugal-type rotary pump (Levitronix LLC) was implanted.

During ICU stay, 21 patients (40%) experienced postoperative maximal tSOFA score ≥ 11, taken into account as severe MOFS associated with elevated mortality rates [13]. Among these patients, 13 (62% of this group) died because of MOFS as primary or secondary cause of death, in a median of 13 (11–25) days. In particular, 3 patients suffered of hepatic injury, 3 suffered of intestinal ischemia, 2 showed a nonresponsive vasoplegia, 3 had respiratory failure, 1 was affected by major esophagus bleeding, and 1 was affected by disseminated intravascular coagulation. The other 32 experienced moderate MOFS with postoperative maximal tSOFA score < 11; they were all alive at 3 months after intervention.

Retrospectively, MCS-candidates were divided in 3 groups according to severity grade of post-operative MOFS and 3-month outcome. Group A: consisting of 32 patients with maximal post-operative tSOFA score < 11 and without adverse events; group B: 8 patients with favorable outcome at 3 months but with maximal postoperative tSOFA score ≥ 11; and group C involving the other 13 patients with and tSOFA score ≥ 11 and adverse lethal event. Detailed pre-implant characteristics and peri-operative data of these 3 groups are described in Table 1. Age, aetiology and Interagency Registry for Mechanically Assisted Circulatory Support (INTERMACS) profiles were comparable among groups as well as the medical therapies. The LVEF and CI values are the only echocardiographic and hemodynamic parameters that differ among A, B, and C groups. The tSOFA score and eGFR values were different among groups, in particular eGFR levels of B and C groups were lower than those of A group. Variables related to surgery as well as type of MCS devices used (data not shown) were comparable among groups.

### 3.1. Preimplant Inflammatory Status

At preimplant, the levels of proinflammatory (TNF-*α*, IL-6, IL-8) and anti-inflammatory (IL-10 and IL-1ra) cytokines, and adhesion molecules (sICAM-1 and sP-selectin), Neopterin/Creatinine ratios and CRP were similar among groups (Table 2).

### 3.2. Preimplant Clinical Variables Associated with Moderate MOFS

All the variables of Tables 1 and 2 that reached a* P* value <0.05 were entered into the multivariable ordinal logistic regression analysis. The only variables independently associated to group A were LVEF and eGFR levels (LVEF: OR 0.847, 95% CI 0.734–0.977, *P* = 0.024; eGFR: OR 0.936, 95% CI 0.898–0.975, *P* = 0.001).

### 3.3. Postoperative Hemodynamic, tSOFA Score, and CRP Profiles

Postoperative recovery and hemodynamic parameters were similar among groups and were maintained during MCS.

**Figure 1:**
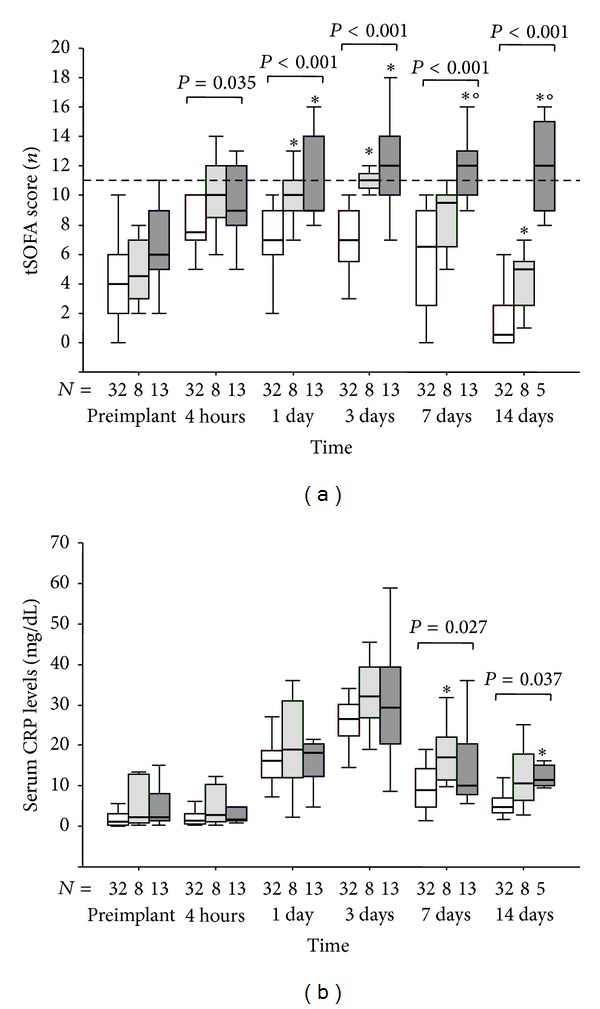
(a) Time course of the t-SOFA score and (b) CRP levels in patients that experienced postoperative maximal tSOFA score < 11 (group A: empty box-plots), and patients with postoperative maximal tSOFA score ≥ 11, with positive (group B: light gray box-plots) or negative (group C: dark gray box-plots) 3-month outcome. The tSOFA score ≥ 11 is pointed out by a dashed line. *P* values are for differences among groups at each time-point by Kruskall-Wallis text. ∗*P* < 0.05 versus group A by Mann-Whitney text corrected by Bonferroni. °*P* < 0.05 versus group B by Mann-Whitney text corrected by Bonferroni.

At 1–3 postoperative days (Figure 1(a)), t-SOFA score was significantly higher in patients of B and C groups compared to A group, while at 7 and 14 days, this parameter remained higher only in C group compared to the others.

Serum levels of CRP were similar among group till 3 days after intervention (Figure 1(b)) but they significantly increased in B group compared to A group at 7 days postimplant. At 14 days, only patients of C group showed higher CRP levels with respect to A group while no difference was instead observed between B and C groups.

### 3.4. Postoperative Profiles of Proinflammatory Cytokines and Neopterin

**Figure 2:**
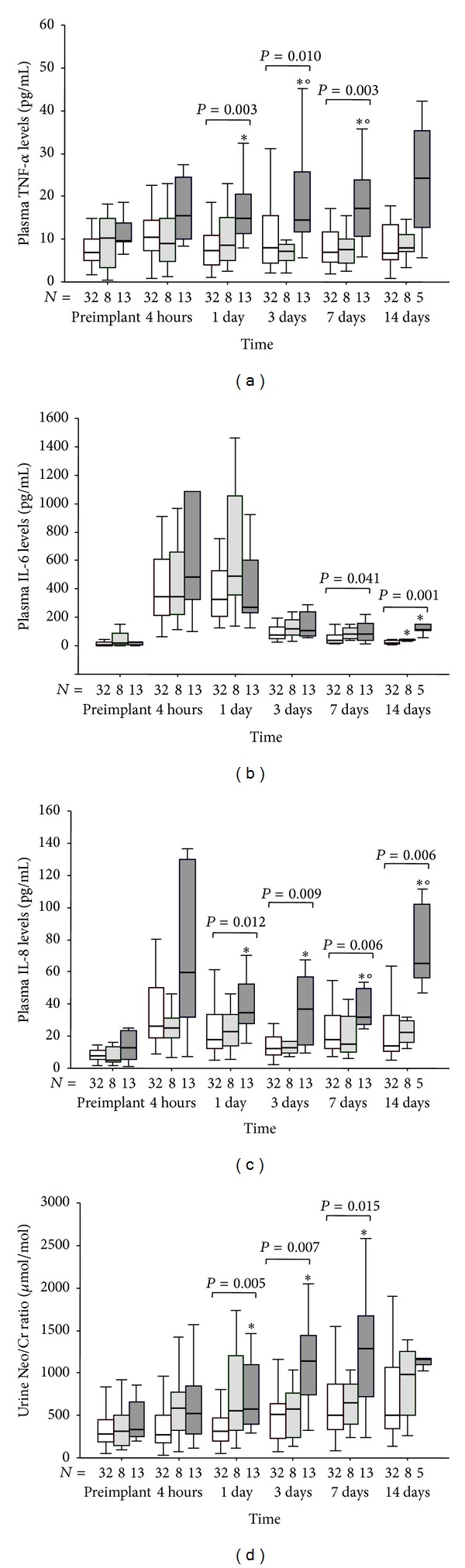
(a) Time course of plasma TNF-*α*, (b) IL-6, (c) IL-8 levels, and (d) urine neopterin/creatinine ratio in patients that experienced postoperative maximal tSOFA score < 11 (group A: empty box-plots), and patients with postoperative maximal tSOFA score ≥ 11, with positive (group B: light gray box-plots) or negative (group C: dark gray box-plots) 3-month outcome. *P* values are for differences among groups at each time-point by Kruskall-Wallis text. ∗*P* < 0.05 versus group A by Mann-Whitney text corrected by Bonferroni. °*P* < 0.05 versus group B by Mann-Whitney text corrected by Bonferroni.

At 1 postoperative day (Figure 2(a)), TNF-*α* levels significantly increased in patients of C group with respect to A group, while after 3 and 7 days the TNF-*α* concentrations of C group were higher compared to both A and B groups. IL-6 levels (Figure 2(b)) differed among groups at 7 days but also at 14 days with B and C groups having higher IL-6 concentrations than those of A group.

At 1 and 3 postoperative days, IL-8 levels (Figure 2(c)) were significantly increased in C group than in A group while at 7 and 14 days, the IL-8 concentrations of C group were also significantly higher compared with those of B group.

In regard to neopterin, an established marker of monocyte activation, we observed an increase of its concentration in patients of C groups with respect to A group at 1, 3, and 7 days (Figure 2(d)).

No significant differences of TNF-*α*, IL-8, and neopterin levels at each postoperative point were observed between A and B groups.

### 3.5. Postoperative Profiles of Anti-Inflammatory Cytokines

**Figure 3:**
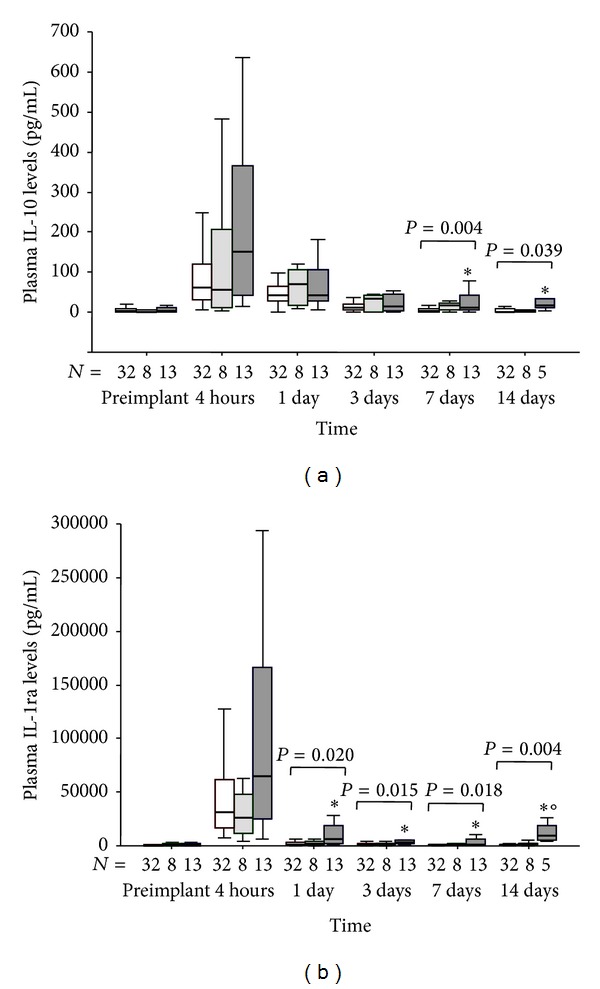
(a) Time course of plasma IL-10 and (b) IL-1ra levels in patients that experienced postoperative maximal tSOFA score < 11 (group A: empty box-plots), and patients with postoperative maximal tSOFA score ≥ 11, with positive (group B: light gray box-plots) or negative (group C: dark gray box-plots) 3-month outcome. *P* values are for differences among groups at each time-point by Kruskall-Wallis text. ∗*P* < 0.05 versus group A by Mann-Whitney text corrected by Bonferroni. °*P* < 0.05 versus group B by Mann-Whitney text corrected by Bonferroni.

The IL-10 levels (Figure 3(a)) were significantly different in C group than in A group at 7 and 14 days while the IL-1ra concentrations (Figure 3(b)) were different between C and A groups of patients at 1, 3, 7, and 14 days after intervention but also with B group at 14 days.

### 3.6. Postoperative Profiles of Adhesion Molecules

**Figure 4:**
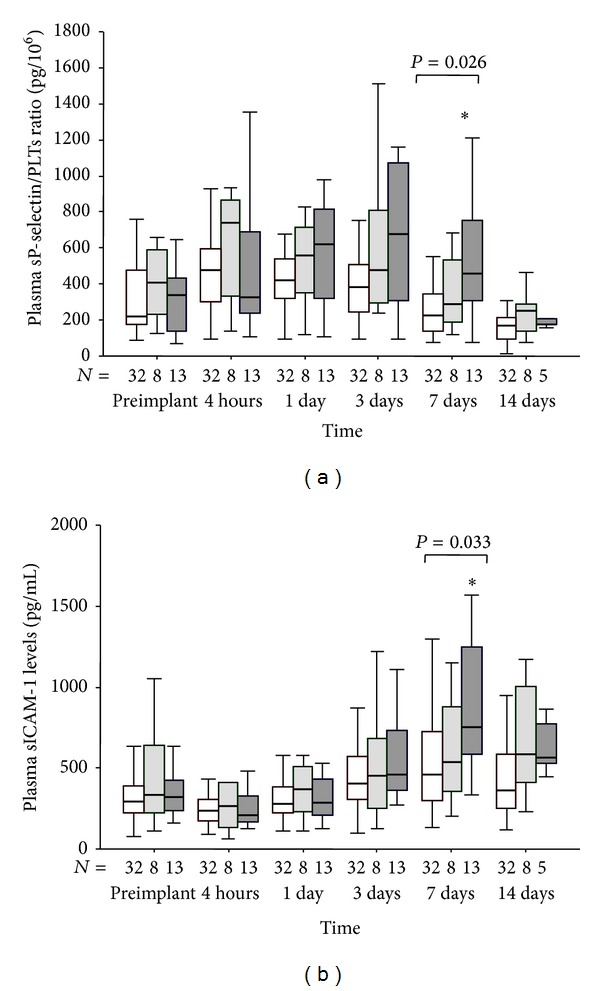
(a) Time course of plasma sP-selectin normalized by platelet (PLT) count and (b) sICAM-1 levels in patients with postoperative maximal tSOFA score < 11 (group A: empty box-plots), and patients with postoperative maximal tSOFA score ≥ 11 and positive (group B: light gray box-plots) or negative (group C: dark gray box-plots) 3-month outcome. *P* values are for differences among groups at each time-point by Kruskall-Wallis text. ∗*P* < 0.05 versus group A by Mann-Whitney text corrected by Bonferroni. °*P* < 0.05 versus group B by Mann-Whitney text corrected by Bonferroni.

sP-selectin o and sICAM-1 levels (Figures 4(a) and 4(b)) were increased in patients of C group than in those of A group at 7 days post device implant.

No significant differences of sP-selectin and sICAM-1 levels were instead observed between A- and B-groups.

### 3.7. Postoperative IL-8 and TNF-*α* Exposure according to Patient Groups

**Figure 5:**
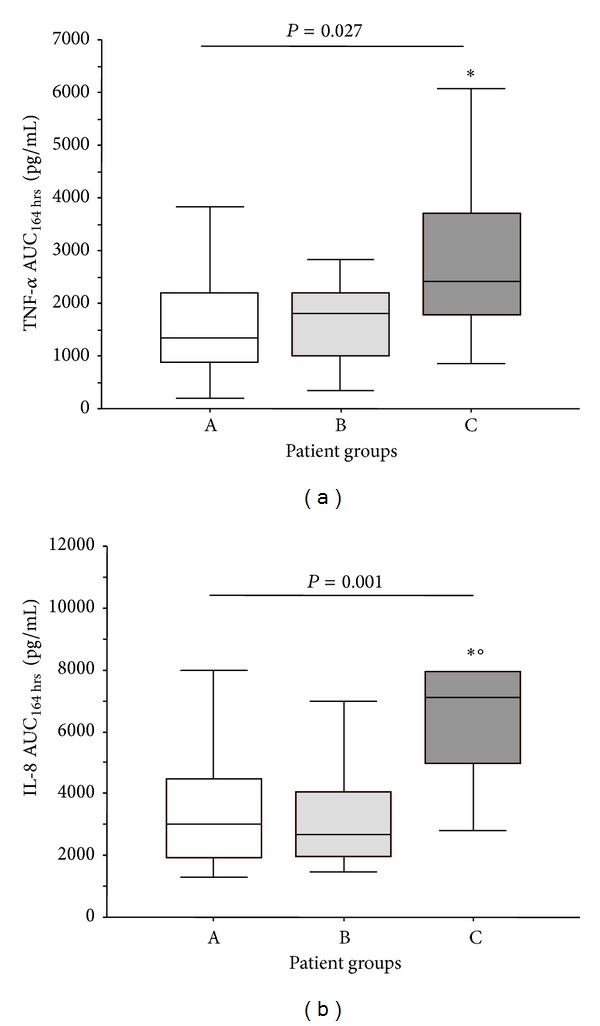
(a) The area under the curve of TNF-*α* and (b) IL-8 levels from 4 hours at 1 week after intervention (AUC_164 hrs_) in patients that experienced postoperative maximal tSOFA score < 11 (group A: empty box-plots), and patients with postoperative maximal tSOFA score ≥ 11, with positive (group B: light gray box-plots) or negative (group C: dark gray box-plots) 3-month outcome. *P* values are for differences among groups by Kruskall-Wallis text. ∗*P* < 0.05 versus group A by Mann-Whitney text corrected by Bonferroni. °*P* < 0.05 versus group B by Mann-Whitney text corrected by Bonferroni.

The grade of patient exposure to TNF-*α* and IL-8 during the first postoperative week was measured calculating the area under the curve of respective levels of cytokines from 4 hours at 1 week (equal to 164 hours) after intervention (AUC_164 hrs_) by trapezoidal rule. The AUC_164 hrs_ of TNF-*α* and IL-8 were differently distributed among patient groups (Figure 5). In particular, the TNF-*α*  AUC_164 hrs_ of C group was higher with respect to A group, while the IL-8 AUC_164 hrs_ of C group was higher than those of A and B groups of patients (Figures 5(a) and 5(b)).

The AUC_164 hrs_ of TNF-*α* and IL-8 of B group were comparable to those of A group.

## 4. Discussion

This study investigated the clinical characteristics and inflammatory mechanisms associated to evolution of severe multiorgan dysfunction in patients affected by ESHF after MCS device implantation. The study shows that ESHF patients, with preoperative reduced LVEF and eGFR values, are more susceptible to develop a severe MOFS and thus an unfavorable outcome after MCS intervention. During the first postoperative week, the grade of inflammatory response, particularly linked to early IL-8 and TNF-*α* exposure, is associated to the clinical evolution of patients with severe MOFS.

The application of MCS has become an effective therapeutic option for treatment of deteriorating phase of HF. However MOFS remains a frequent early complication in MCS-treated patients, and an adequate knowledge of the mechanisms involved in its onset and deterioration are still lacking. Several studies reported that, after cardiac surgery, the grade of MOFS severity developed in the first days of ICU stay and is associated to the clinical outcome. High tSOFA scores are proposed as useful outcome predictors [13]; independently of the starting value, a tSOFA score ranging from 8 to 11 during the first 48 hours in the ICU, is associated to a mortality rate of 60 to 90%. In the present study, we used an increase in tSOFA scores ≥ 11 during the first weeks in ICU as threshold value to distinguish MCS patients developing a severe MOFS from those that experienced a moderate multiorgan damage (tSOFA score < 11). This threshold score has been able to discriminate also in our study patients at risk to unfavorable outcome. In fact, the mortality rate of subjects with tSOFA score ≥ 11 was 62%.

tSOFA value between patients of B and C groups was comparably high until the third day postintervention but decreased in B group at 7 and 14 days compared to C group, suggesting that this period may be crucial for the clinical evolution of these critical patients.

One day postintervention, the levels of cytokines, TNF-*α* and IL-8, and the urinary concentrations of neopterin significantly increased in C group compared to A and B groups. Moreover, during the first postoperative week, C group of patients showed a greater exposure to TNF-*α* and IL-8 with respect to the other 2 groups. TNF-*α* is known to be involved in systemic inflammatory response syndrome (SIRS), which usually precedes MOFS in several ill patients [14, 15], while IL-8 is a specific monocyte attracting chemokines that modulates monocyte activation, an important condition in organ damage and haemostatic complications [16]. These data suggest that, immediately after MCS intervention, the extent of inflammatory response related mainly to TNF-*α* and IL-8 signals could influence the development and degree of MOFS severity, contributing to subsequent clinical evolution of ESHF patients.

On the contrary, CRP levels, the main inflammatory variable routinely used in the setting of MCS-therapy, were not able to early discriminate patients with unfavourable outcome, suggesting that CRP profile does not reflects the inflammatory pathways involved in the adverse evolution of severe MOFS.

During the early phase of MCS, the exacerbate release of anti-inflammatory cytokines (IL-10 and IL-1ra) in addition to a massive proinflammatory reaction, could produce an unbalance of inflammatory response which might contributes to the onset of severe MOFS by causing anergy and immunosuppression [17], a condition known as immunoparalysis in patients with severe sepsis [18].

One postoperative week, patients with unfavorable evolution of severe MOFS showed also elevated sP-selectin and adhesion molecule sICAM while the neopterin levels of these patients already increased 1 day after surgery. sP-selectin is a marker of platelet and endothelial alteration and a direct inducer of procoagulant activity associated with thrombotic disease [19, 20]. sICAM is responsible for neutrophil and leukocyte attachment to the endothelium [19] while neopterin is a marker of macrophage/monocyte and immune system activation. The activation of monocyte and the injury of endothelium are factors that might contribute, in the microcirculation, to the formation of microthrombi and intravascular coagulation associated to development and worsening of multiorgan failure [7].

In previous studies, involving MCS-treated patients with left ventricular and biventricular supports, several preimplant clinical characteristics such as cardiogenic shock, advanced age, severe right heart failure, or increased bilirubine, were found to be predictors of adverse outcomes [21, 22]. In our series, patients characterized by lower LVEF values and altered renal function before device implantation, developed serious MOFS with unfavourable evolution after MCS intervention. On the contrary, preimplant inflammatory mediators did not differ among groups, indicating that this mechanism is activated during or immediately after surgery. The intensity and length of postimplant inflammatory response seems to be an important trigger that influences the clinical outcome of these patients.

Several studies have recently highlighted the emergent role of specialized regulatory cell subtypes belonging to myeloid-derived suppressor, monocyte phenotypes, and T and B lymphocytes in the proresolution of inflammatory pathways in critically ill patients [23, 24]. The evaluation of these subtypes of inflammatory cells in subjects' candidates to MCS therapy could contribute to identify specific linkages between cellular inflammatory status and unbalanced inflammatory response associated to unfavorable outcome.

## 5. Conclusions

A specific inflammatory sequence, involving inflammatory signals dependent from TNF-*α* and IL-8, monocyte, and endothelial activation, contributes to the unfavourable evolution of severe MOFS developed during the first weeks after MCS therapy. Patients, characterized by preimplant low ejection fraction and renal dysfunction, are more susceptible to develop an unbalanced inflammatory response during MCS therapy associated to a severe MOFS with negative outcome. High TNF-*α*, IL-8, and neopterin concentrations may be considered as early markers of MOFS occurrence and unfavourable outcome in end stage HF patients. The correct timing to MCS implant is crucial to prevent the excessive clinical deterioration after MCS implantation triggered by an aberrant postoperative inflammatory response, which adversely affects the clinical outcome of MCS therapy.

## Figures and Tables

**Table 1:** Clinical characteristics of patients and perioperative data.

	All cases	max tSOFA score < 11	max tSOFA score ≥ 11		*P*
	(*n* = 53)	(Group A = 32)	Outcome + (group B = 8)	Outcome − (group C = 13)	
Age, years	54 (48–61)	52 (45–58)	53 (49–62)	57 (52–66)	0.052
Male gender, *n* (%)	49 (93)	31 (97)	7 (88)	11 (85)	0.313
INTERMACS profile, *n* (%)					0.610
1	15 (28)	8 (25)	2 (25)	5 (38)	
2	15 (28)	10 (31)	2 (25)	3 (23)	
3	22 (42)	14 (44)	4 (50)	4 (31)	
4	1 (2)	—	—	1 (8)	
Etiology, *n* (%):					0.318
DCM	30 (57)	20 (63)	5 (63)	5 (39)	
ICM	22 (41)	12 (37)	3 (37)	7 (53)	
Myocarditis	1 (2)	—	—	1 (8)	
MAP, mmHg	75 (70–85)	78 (72–85)	75 (69–83)	73 (68–83)	0.499
LVEF, %	21 (18–26)	23 (18–26)	26 (19–28)	20 (16–21)	0.045
LVEDV, mL	260 (190–312)	260 (190–330)	239 (174–326)	237 (161–292)	0.466
RAP, mmHg	6 (4–10)	5 (3–8)	8 (4–15)	9 (4–16)	0.163
PCWP, mmHg	24 (18–30)	24 (15–30)	23 (10–33)	24 (19–31)	0.865
CI, L/min/m^2^	1.69 (1.36–2.00)	1.70 (1.51–2.08)	1.32 (1.07–1.47)∗	1.69 (1.45–1.83)	0.023
Treatments, *n* (%)					
ACEi + ATII	34 (67)	21 (68)	5 (63)	8 (67)	0.961
Beta-blocker	31 (61)	20 (65)	6 (75)	5 (42)	0.259
Statins	13 (27)	9 (30)	2 (25)	2 (18)	0.745
Diuretics	39 (77)	25 (81)	7 (88)	7 (58)	0.219
Inotropic	37 (71)	23 (72)	5 (63)	9 (75)	0.824
Inotropic equivalents, *n*	5 (0–10)	4 (0–9)	4 (0–8)	9 (1–13)	0.335
IABP, *n* (%)	22 (43)	13 (42)	2 (25)	7 (58)	0.329
WBC, 10^9^/L	8.4 (7.1–11.4)	8.8 (7.2–11.9)	8.5 (5.6–17.2)	8.4 (6.5–9.4)	0.580
INR	1.2 (1.1–1.4)	1.2 (1.0–1.5)	1.2 (1.1-1.2)	1.2 (1.1–1.4)	0.407
Lactate, mg/dL	1.0 (0.8–1.8)	1.2 (0.8–1.7)	1.0 (0.7–1.8)	1.0 (0.8–2.8)	0.814
eGFR, mL/min/1.73 m^2^	80 (58–100)	86 (74–116)	57 (50–76)∗	59 (42–76)∗	0.001
t-Bil, mg/dL	1.04 (0.63–1.86)	0.98 (0.61–1.89)	1.28 (0.89–2.37)	0.84 (0.57–1.91)	0.482
tSOFA score, *n*	5 (3–7)	4 (2–6)	5 (3–7)	6 (5–10)	0.051
Perioperative data					
Surgery time, min	310 (255–375)	308 (270–368)	340 (308–383)	275 (213–390)	0.312
CPB time, min	83 (68–102)	81 (65–104)	88 (68–122)	84 (72–96)	0.828

Data are expressed as median and interquartile range (25th–75th) or number (percentage).

ACEi: angiotensin converting enzyme inhibitor; t-Bil: total Bilirubin; BUN: blood urea nitrogen; CI: cardiac index; CPB: cardiopulmonary bypass; DCM: dilated cardiomyopathy; ECMO: extracorporeal membrane oxygenation; eGFR: estimated glomerular filtrate rate; IABP: intra-aortic balloon pump; ICM: ischemic cardiomyopathy; INR: international normalized ratio; INTERMACS: Interagency Registry for Mechanically Assisted Circulatory Support; LVEF: left ventricular ejection fraction; LVEDV: left ventricular end-diastolic volume; MAP: mean arterial pressure; PCWP: pulmonary capillary wedge pressure; RAP: right atrial pressure; tSOFA: total sequential organ failure assessment; WBC: white blood cell count; +: positive; −: negative.

*P < 0.05 versus group A patients by Bonferroni post-hoc test.

**Table 2:** Preimplant inflammatory characteristics.

	All cases	max tSOFA score < 11	max tSOFA score ≥ 11		*P*
	(*n* = 53)	(Group A = 32)	Outcome + (group B = 8)	Outcome − (group C = 13)	
TNF-*α*, pg/mL	9.3 (5.2–12.1)	6.9 (5.0–10.4)	10.3 (2.6–16.3)	9.7 (9.4–14.0)	0.081
IL-6, pg/mL	9.8 (3.6–27.4)	6.8 (3.0–24.1)	17.8 (6.1–118.7)	25.6 (9.6–63.4)	0.067
IL-8, pg/mL	7.6 (5.2–13.6)	7.7 (5.5–11.3)	5.1 (3.7–14.4)	13.1 (5.3–24.2)	0.294
C-reactive protein, mg/dL	1.5 (0.5–3.9)	1.2 (0.3–3.1)	2.2 (0.8–13.1)	2.2 (1.1–8.4)	0.108
IL-10, pg/mL	1.8 (0–7.5)	1.8 (0–8.0)	1.7 (0–6.2)	2.2 (0–14.4)	0.824
IL-1ra, pg/mL	518 (311–1276)	491 (295–840)	785 (268–1955)	828 (468–1898)	0.137
sICAM-1, pg/mL	300 (224–433)	291 (226–397)	332 (220–676)	323 (210–528)	0.739
sP-selectin/PLT, pg/10^6^	288 (164–505)	221 (173–476)	410 (217–622)	337 (122–537)	0.742
Neo/Cr ratio, *μ*mol/mol	290 (199–563)	279 (181–457)	317 (128–549)	337 (240–698)	0.422

Data are expressed as median and interquartile range (25th–75th).

IL: interleukin; Neo/Cr: neopterin levels normalized by urine creatinine levels; sICAM-1: soluble form of intercellular adhesion molecule type 1; sP-selectin/PLT: soluble platelet selectin normalized by platelet count; TNF: tumor necrosis factor; +: positive; −: negative.

## Abbreviations

CRP: C-reactive protein
eGFR: Estimated glomerular filtration rate
ESHF: End-stage heart failure
HF: Heart failure
IL-: Interleukin
INTERMACS: Interagency Registry for Mechanically Assisted Circulatory Support
LVEF: Left ventricular ejection fraction
MCS: Mechanical circulatory support
MOFS: Severe multiorgan failure syndrome
sICAM-1: Soluble intercellular adhesion molecule type 1
sP-selectin: Soluble platelet selectin
TNF: Tumor necrosis factor
tSOFA: Total sequential organ failure assessment score.
